# Selections

**Published:** 1893-02

**Authors:** 


### Selections.

### USE OF IODINE, CARBOLIC ACID, AND CHLORAL IN SKIN DISEASES.

Dr. C. W. Cutler (*Journal of Cutaneous and Genito-Urinary Diseases*) says:

After two years experience in the use of this therapeutic agent, I believe the following conclusions can be safely drawn:

That we have in this combination of chloral, carbolic acid, and tincture of iodine, in equal portions, a topical remedy of decided value for the treatment of certain affections of the skin.

That the combination of these agents produces better results, has a wider range of usefulness, and possesses superior therapeutic advantages than are found in either of the remedies when employed alone.

That the physiological properties of this solution, upon which the therapeutic advantages of the remedy depend, are those of an antiseptic, antipruritic, antiparasitic, antiphlogistic, analgesic, anæsthetic, absorbent, and counter-irritant nature.

That the solution is a powerful agent and should not be used indiscriminately or carelessly, as there is danger of producing severe dermatitis and constitutional poisoning.

That its chief therapeutic advantages are due to its penetrating action into the tissues of the skin, its rapid destruction of all forms of micro-organisms, and its wonderful power in hastening the absorption of inflammatory products.

That it is, therefore, especially serviceable in parasitic skin affections and in all forms of chronic skin diseases characterized by thickening and induration of the skin, accompanied by scaling and itching.

That it changes the form of some skin diseases, substituting for the original disease an acute dermatitis, which responds readily to treatment.—*American Lancet.*

